# Efficacy of lotilaner (Credelio™) against the adult cat flea, *Ctenocephalides* *felis* and flea eggs following oral administration to dogs

**DOI:** 10.1186/s13071-019-3873-1

**Published:** 2020-01-14

**Authors:** Lisa Young, Daniela Karadzovska, Scott Wiseman, Rainer Helbig

**Affiliations:** 1 Elanco Animal Health, 2500 Innovation Way, Greenfield, IN 46140 USA; 2 Elanco Australasia Pty Ltd, 245 Western Road, Kemps Creek, NSW 2178 Australia; 3 Elanco Animal Health, Lilly House, Priestly Road, Basingstoke, Hants RG24 9NL UK; 4 Elanco Animal Health, Mattenstrasse 24a, 4058 Basel, Switzerland

**Keywords:** Lotilaner, Credelio, Fleas, Dog, Efficacy, Flea egg production, Prevention

## Abstract

**Background:**

A blinded, randomized, negative controlled laboratory study was conducted to evaluate the efficacy of lotilaner (Credelio^TM^, Elanco) when administered orally to dogs, against experimentally induced adult flea infestations and flea egg production.

**Methods:**

Twenty dogs were selected for the study and allocated to two treatment groups. Ten dogs were treated with lotilaner (at the lower half of the recommended dose range of 20–43 mg/kg) on Day 0. Ten dogs treated with placebo tablets served as the control group. Each dog was infested with 100 unfed adult *C. felis* fleas on days -1, 6, 13, 20 and 29. At 24 h post-treatment or post-infestation, each dog was combed for the removal and counting of adult live fleas. Flea eggs were also collected and counted from the pan under each dog cage.

**Results:**

Dogs in the lotilaner treated group received a mean dose of 22.6 mg/kg (range 20.2–25.9 mg/kg) and no adverse events were observed in any dog in this study. At each evaluation time point, the lotilaner group provided 100% efficacy against adult live flea counts as compared to the placebo control group. Egg production from lotilaner treated dogs was reduced by 98.5% (geometric mean; 97.4% arithmetic mean) 24 h post-treatment (and 48 h post-flea infestation). No eggs (100% efficacy) were available for collection following infestations on Day 6 onwards from the lotilaner treated dogs. At each evaluation time point, adult live flea counts from the lotilaner treated dogs were significantly lower (*P* < 0.0001) than from the placebo control group.

**Conclusions:**

In dogs treated with a single dose of lotilaner (mean dose 22.6 mg/kg), 100% of adult fleas were killed within 24 h post-treatment or post-subsequent infestations as compared to the placebo control group, thereby demonstrating that lotilaner kills fleas before they can lay eggs thus preventing subsequent flea infestations for 30 days after treatment. There were no reported adverse events in any dogs, demonstrating that lotilaner tablets were well tolerated at the dose rates assessed in this study.
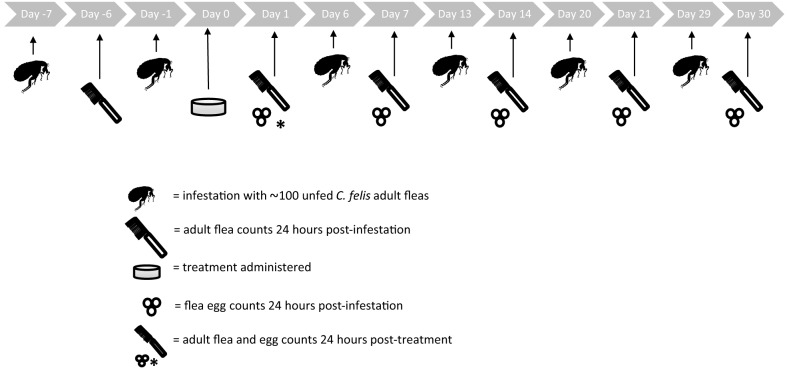

## Background

The adult cat flea, *Ctenocephalides felis felis*, is the most prevalent flea species worldwide. It is one of the four known subspecies of *C. felis* but lacks the geographical restrictions of the other three subspecies; *C. felis strongylus* and *C. felis darmarensis* are found in Africa, and *C. felis orientis* is found in Southeast Asia and the East Indies [1]. In this article, the use of the terms flea and *C. felis* refers to C. *felis felis*.

Adult fleas are the only life stage commonly found infesting domestic cats and dogs worldwide [1]. Within 24 to 36 h of acquiring a host and feeding, female fleas begin laying their eggs [2, 3]. An infestation rapidly follows as each female flea is capable of producing as many as 50 eggs per day [1]. Flea eggs drop off the host into the environment and within a few weeks develop into adult fleas [2]. In the absence of effective treatments, these stages continue to develop and cause an increasing flea burden.

For a flea control program to be considered successful, it should minimize a dog’s exposure to the flea’s salivary antigens, reduce the time of flea feeding as well as the risk of pathogens’ transfer from flea to host and prevent egg laying, thus contributing to a depletion of the flea stages in the dog’s environment [1]. Despite several decades of widely available flea control products, infestations remain a health concern for companion animals, an issue compounded by the presence of insecticide-resistant populations of *C. felis* [4].

The isoxazoline class represents the newest parasiticides marketed for the treatment of flea and tick infestations in dogs. These compounds are available as oral or topical formulations and present a broad spectrum of antiparasitic activity [5]. The isoxazolines are potent inhibitors of the gamma-aminobutyric acid-gated chloride channels, resulting in rapid death of fleas and ticks after oral administration to dogs [6–8].

Lotilaner is the newest isoxazoline compound approved for the treatment of flea and tick infestations in dogs (Credelio™ chewable tablets for dogs; Elanco, Greenfield, IN, USA) [9]. A pharmacokinetic study has shown that, when administered with food, lotilaner is rapidly absorbed, with peak plasma concentrations occurring within approximately 2 h following oral administration, and a half-life of approximately 30 days [10]. The rapid absorption of lotilaner translates into a rapid onset of activity against fleas with laboratory studies showing lotilaner begins killing fleas and ticks within 4 h after treatment [11, 12]. Efficacy against fleas and ticks was then sustained through 35 days after a single treatment, indicating that monthly use of lotilaner is effective in reducing the flea life-cycle stages in a dog’s environment [13]. Additionally, preclinical studies demonstrated lotilaner to be safe when administered monthly over eight consecutive months at the highest dose range (20 to 43 mg/kg), and at three- and five- times that dose to puppies from 8 weeks of age [14].

In this study, the authors evaluated the efficacy (against adult fleas and egg production) and safety of lotilaner administered once, at the lower half of the registered dose range (corresponding to 20–31.5 mg/kg), to dogs experimentally infested with fleas. A placebo control tablet was used as the negative control.

## Methods

This study was a blinded, randomized, negative controlled laboratory study. Blinding of the study was achieved through the separation of functions. All personnel conducting observations, performing flea infestations and carrying out flea counts were blinded to treatment allocation.

### Animals

Twenty purpose bred Beagle or mixed breed dogs aged between 13 to 28 months were used for this study. All dogs demonstrated good flea retention levels prior to inclusion into the study; none of the dogs had been treated with an ectoparasiticide for at least 12 weeks, and all were in good health at the start of the acclimation phase. Dogs were individually housed in runs that conformed to accepted animal welfare guidelines. During flea infestation, dogs were housed in individual metabolism cages to facilitate accurate collection and enumeration of eggs and to prevent cross-contaminations. Dogs were fed an appropriate maintenance ration of a commercial dog food. Water was available *ad libitum*. Dogs were observed for general health at least once daily throughout the study.

### Animal selection and randomization

On Day -7, 26 dogs were infested with 100 unfed adult fleas (*C. felis*; assumed to be approximately 50% male and 50% female) of a laboratory bred colony (USA strain). On Day -6, flea comb counts were performed to assess the susceptibility of each dog to maintain experimental infestations and for random allocation of dogs to treatment groups. Flea combing using a fine toothed combed was completed in accordance to the laboratory’s standard operating procedure. The 20 dogs with the highest pre-treatment live adult flea counts were used in this study. To ensure the treatment groups were balanced with respect to the flea retention pre-treatment live adult *C. felis* infestation levels were used as the blocking factor. Ten blocks of 2 dogs were defined and within each block, each treatment was represented once.

### Treatments

On the day of treatment, each dog was offered approximately 1/3 of the manufacturer’s recommended daily amount of a palatable canned dog food, based on the dog’s weight. All dogs were given up to 30 min to eat the offered food. Once a given dog had eaten all of the food offered, then that dog was dosed immediately. Treatment was based on the assigned treatment group and the Day -6 body weight. Single or multiple combinations of lotilaner tablet sizes were used to achieve dosing in the lower half of the recommended dose range (corresponding to 20–31.5 mg/kg). The placebo control tablet was a vehicle control tablet containing no lotilaner and equivalent in size to the lotilaner tablets. Following dosing, each dog was offered their standard daily maintenance diet. Dogs were observed following treatment for any adverse events.

### Efficacy evaluations

**Fig. 1:**
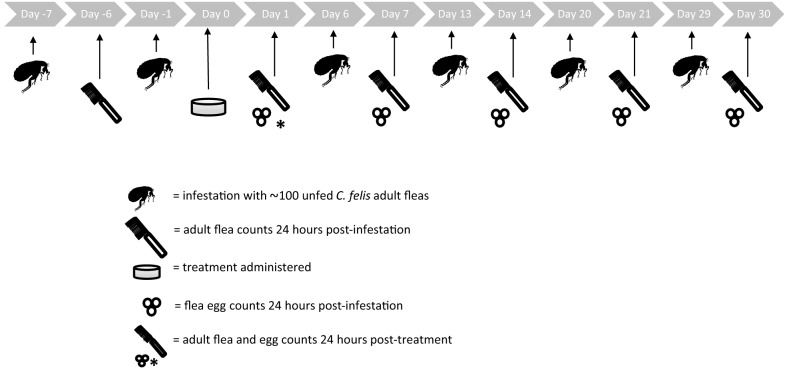
Timings of infestations, counts and treatment

On Days -1, 6, 13, 20 and 29, each dog was infested with approximately 100 unfed adult fleas (*C. felis*; approximately 50% male and 50% female; Fig. 1). The fleas used for all infestations were from a laboratory bred colony (USA strain) of *C. felis*. At each infestation, adult unfed fleas were placed along the dorsal midline of each animal, from its head to the base of its tail.

Dogs were transferred to temporary housing (metabolism cages) on Day 0, immediately after treatment administrations, and on Days 6, 13, 20 and 29 when new flea infestations were performed. Individual housing was maintained until fleas were counted and disposed.

All adult fleas were removed and counted from each dog on Days 1, 7, 14, 21 and 30 at 24 h post-dosing or 24 h post-infestation (Fig. 1). The number of live fleas on each dog was recorded, where a live flea was defined as a flea exhibiting normal behavior or with any response to external stimuli.

Flea eggs were collected from the collection pan under each cage at these same time points and separated from each dog’s debris (e.g. hair, food, etc.) before counting.

For each counting day, the study statistician randomized the order in which the dogs had their adult flea and flea egg collections and counts performed.

After counting, the dog was returned to its normally assigned run.

### Data analysis

At each assessment time point, percent efficacy was calculated using arithmetic and geometric means with Abbott’s formula:

$${\text{Efficacy }}\left( \% \right) \, = \, \left( {\left( {{\text{C }} - {\text{ T}}} \right) \, /{\text{ C}}} \right) \, \times { 1}00$$

where C is the geometric/arithmetic mean number of adult live fleas in the placebo control group and T is the geometric/arithmetic mean number of adult live fleas in the lotilaner treated group

Geometric means involved taking the logarithm of the flea count of each animal, flea counts that were equal to zero had a one added to the count for every animal in each group. Since one was added to the flea count of every animal, the constant (1) was subtracted from the resultant calculated geometric mean prior to calculating the percent effectiveness with the geometric mean. The percent efficacy on egg production was also calculated using Abbott’s formula.

The log-transformed flea count data were analyzed using a mixed linear model that included treatment group as a fixed effect and block as a random effect. Additionally, non-parametric methods were used to compare the lotilaner group with the placebo control group (Mann-Whitney U-test) to allow for the possibility that the assumption of normally distributed model residuals were not met.

All analyses were performed using SAS/STAT^®^ software (Version 9.3, SAS Institute Inc., Cary, NC, USA) with the level of significance of the formal tests was set at 5%; all tests were two-sided.

## Results

Dogs in the placebo control group and lotilaner treated group weighed an average of 9.74 kg and 10.51 kg, respectively (Table 1).

**Table 1:** Demographics of study animals

Variable	Statistic	Placebo control (*n* = 10)	Lotilaner (*n* = 10)
Age (months)	Mean ± SD	15.9 ± 3.7	19.1 ± 5.5
	Range	13–25	13–28
Weight (kg)	Mean ± SD	9.74 ± 1.51	10.51 ± 2.10
	Range	7.40–12.20	7.00–13.60
Breed			
Beagle	*n* (%)	5 (50)	3 (30)
Mixed breed	*n* (%)	5 (50)	7 (70)
Hair coat length			
Short	*n* (%)	10 (100)	10 (100)
Sex			
Male	*n* (%)	3 (30)	6 (60)
Female	*n* (%)	7 (70)	4 (40)
Dose (mg/kg)	Mean ± SD	0	22.6 ± 1.8

All dogs included in the study demonstrated adequate pre-treatment flea retention with Day -6 flea counts ranging from 78–100 fleas/dog and geometric means of 91.0 (range 81–100; 91.3% arithmetic mean) in the placebo control group and of 91.0 (range 78–100; 91.2% arithmetic mean) in the lotilaner treated group. Adequate flea infestations were obtained in all 10 dogs in the placebo control group at each post-treatment evaluation time point with average (arithmetic mean) infestation rates ranging from 87.8 to 93.1 % (Table 2).

**Table 2:** Adequacy of infestation of live adult fleas in placebo control dogs

Day	No. of adequately infested dogs/total no. of dogs	Average infestation (%)
1	10/10	91.4
7	10/10	92.6
14	10/10	92.9
21	10/10	93.1
30	10/10	87.8

Dogs in the lotilaner treatment group received an average dose range of 22.6 mg/kg (20.2– 25.9 mg/kg; Table 1). This single dose of lotilaner provided 100% efficacy against adult *C. felis* on days 1, 7, 14, 21 and 30 (Table 3). At each evaluation time point, the lotilaner group live flea counts were statistically different (*P* < 0.0001; both parametric and non-parametric statistical models) as compared to the placebo control group.

**Table 3:** Summary of adult flea counts and percent efficacy

Day	No. of dogs flea-free (%)		Arithmetic mean (% Efficacy)		Geometric mean (% Efficacy)		Comparison
	Placebo control (*n* = 10)	Lotilaner (*n* = 10)	Placebo control (*n* = 10)	Lotilaner (*n* = 10)	Placebo control (*n* = 10)	Lotilaner (*n* = 10)	
1	0	10 (100)	91.4	0 (100)	91.2	0 (100)	*t*_(9)_ = 203.12, *P* < 0.0001^a^ *Z* = 4.04, *P* < 0.0001^a^
7	0	10 (100)	92.6	0 (100)	92.1	0 (100)	*t*_(9)_ = 123.10, *P* < 0.0001^a^ *Z* = 4.07, *P* < 0.0001^a^
14	0	10 (100)	92.9	0 (100)	92.5	0 (100)	*t*_(9)_ = 147.43, *P* < 0.0001^a^ *Z* = 4.05, *P* < 0.0001^a^
21	0	10 (100)	93.1	0 (100)	92.8	0 (100)	*t*_(9)_ = 167.53, *P* < 0.0001^a^ *Z* = 4.07, *P* < 0.0001^a^
30	0	10 (100)	87.8	0 (100)	87.5	0 (100)	*t*_(9)_ = 154.22, *P* < 0.0001^a^ *Z* = 4.04, *P* < 0.0001^a^

*Note*: Comparison results of both the parametric and non-parametric tests are presented

^a^Values significantly decreased in comparison to the control group

On Day 1 (24 h post-treatment and 48 h post-flea infestation), flea egg counts in the placebo control group ranged from 281 to 809 with a geometric mean of 494.5 (arithmetic mean of 527.0) flea eggs (Table 4). The lotilaner group flea egg counts on Day 1 ranged from 0 to 48 with a geometric mean of 7.3 (arithmetic mean of 13.9) flea eggs. On days 7, 14, 21 and 30, the placebo control group had 86.4, 95.6, 97.0 and 94.6 geometric mean (139.2, 163.1, 188.0 and 154.5 arithmetic mean) flea eggs produced, respectively. Flea egg production for dogs in the lotilaner group was zero for fleas infested after day 0 treatment for the 30 day period assessed in this study.

**Table 4:** Summary of flea egg counts and percent efficacy

Day	Number of dogs egg-free (%)		Arithmetic mean (% Efficacy)		Geometric mean (% Efficacy)	
	Placebo control (*n* = 10)	Lotilaner (*n* = 10)	Placebo control (*n* = 10)	Lotilaner (*n* = 10)	Placebo control (*n* = 10)	Lotilaner (*n* = 10)
1	0	1 (10.0)	527.0	13.9 (97.4)	494.5	7.3 (98.5)
7	1 (10.0)	10 (100)	139.2	0 (100)	86.4	0 (100)
14	1 (10.0)	10 (100)	163.1	0 (100)	95.6	0 (100)
21	1 (10.0)	10 (100)	188.0	0 (100)	97.0	0 (100)
30	1 (10.0)	10 (100)	154.5	0 (100)	94.6	0 (100)

There were no adverse events reported in this study

## Discussion

In this study, lotilaner tablets for dogs (Credelio^TM^) administered orally in the fed state at the lower half of the recommended dose range (mean dose 22.6 mg/kg) demonstrated 100% efficacy against adult fleas for up to 30 days. Adult flea efficacy of 100% was achieved within 24 h post-treatment with lotilaner. Previous studies have confirmed that a single oral treatment with lotilaner starts killing fleas within 4 h of treatment and post-infestation [13]. At 6 h post-treatment/infestation, efficacy against adult fleas was > 99% for up to 35 days [13]. The rapid onset of lotilaner was further shown in a speed of kill study using *C. felis*, where at 1 h post-dosing 22 moribund fleas were collected from five lotilaner treated dogs compared to one moribund flea from a single dog in the control group [11]. A moribund flea was a flea that was laterally recumbent; could not normally move through hair or right itself when placed on a flat surface but still had leg movement or twitching. By 2 h post-dosing, the numbers of moribund fleas had increased substantially with significantly fewer live fleas on treated dogs than the control group [11].

Lotilaner continued displaying excellent adult flea efficacy (100%) in dogs experimentally infested with *C. felis* under laboratory conditions on days 7, 14, 21 and 30 post-infestation. Such efficacy significantly reduced flea egg production by killing fleas before they can lay eggs. Within 24 h of lotilaner administration, egg production almost completely halted (98.5% geometric mean reduction; 97.4% arithmetic mean). At this evaluation time point, control dogs had 494.5 flea eggs (geometric mean; 527.0 arithmetic mean) while the lotilaner group had 7.3 flea eggs (geometric mean; 13.9 arithmetic mean) collected. Flea eggs present in the lotilaner group were likely already in production in female fleas at time of treatment, as the first flea infestation was applied 24 h before dosing, and fleas begin to lay eggs 24 to 36 h after they have begun to feed on an acquired host [3]. The high egg count in the control group 24 h post-treatment compared to the other post-infestation time points is most likely due to the first flea infestation being applied 24 h before dosing. Not a single egg was collected from treated dogs for the next 30 days.

The potential for lotilaner to provide environmental flea control has been further demonstrated in client-owned dogs in field studies conducted in the USA [15] and Europe [16]. In both studies, monthly administration of a flavored chewable tablet at a dose of 20–40 mg/kg for 3 consecutive months resulted in consistent reductions of adult flea counts of 99.3, 99.9 and 100% on days 30, 60 and 90 respectively in the USA study and 99.5, 99.9 and 99.8% on days 28, 56 and 84 in the European study.

Eliminating adult fleas prior to egg production [17, 18] is the aim of any successful pet flea control program. Over time, this also results in elimination of fleas from the petʼs environment. Therefore, the speed of flea knockdown from the time of product administration that is reliably maintained through the next scheduled dose is an important consideration in choosing an appropriate flea control product. For dogs living in a flea-infested environment, monthly treatment with lotilaner will rapidly reduce the existing flea burden on the animals and stop the deposition of eggs in the environment. Lotilaner will also kill fleas that emerge from the infested environment and infest the host, for the month-long treatment period. By killing fleas before they can reproduce, lotilaner depletes the flea stages in a dog’s environment.

## Conclusions

A single dose of lotilaner tablets for dogs (Credelio^TM^) administered orally at a minimum dose of 20 mg/kg demonstrated 100% efficacy against existing adult flea infestations and persistent efficacy for 30 days. Such efficacy significantly prevented flea eggs from being produced following new flea infestations up to 30 days after treatment, in the dog’s local environment. There were no reported adverse events in any dogs, demonstrating that lotilaner tablets were well tolerated at the dose rates assessed in this study.

## Data Availability

Data supporting the conclusions of this article are included within the article. Due to commercial confidentiality of the research, data not included in the manuscript can only be made available to *bona fide* researchers, subject to a non-disclosure agreement.
